# A meta-analysis of the relationship between polycystic ovary syndrome and sleep disturbances risk

**DOI:** 10.3389/fphys.2022.957112

**Published:** 2022-09-29

**Authors:** Chaoyu Wang, Tao Huang, Wu Song, Jinru Zhu, Yanhong Liu, Xiaojuan Chen, Xishi Sun, Qinglan Wu, Huimin Chen, Huizhao Liao, Junyan Lin, Xudong Ou, Zhihong Zou, Zhiwei Wang, Zhenzhen Zheng, Kang Wu, Riken Chen

**Affiliations:** ^1^ Department of Respiratory and Critical Care Medicine, The Second Affiliated Hospital of Guangdong Medical University, Zhanjiang, China; ^2^ Department of Respiratory and Critical Care Medicine, Taishan Hospital of Traditional Chinese Medicine, Jiangmen, China; ^3^ Department of Emergency, Affiliated Hospital of Guangdong Medical University, Zhanjiang, Guangdong, China; ^4^ Department of Laboratory, Dongguan Maternal and Child Health Care Hospital, Dongguan, China; ^5^ Department of Gynecology, Taishan Hospital of Traditional Chinese Medicine, Jiangmen, China; ^6^ Medical College, Jiaying University, Meizhou, China; ^7^ Department of Respiratory and Critical Care Medicine, Central People’s Hospital of Zhanjiang, Zhanjiang, China; ^8^ Guangzhou Medical University, State Key Laboratory of Respiratory Disease, National Clinical Research Center for Respiratory Disease, Guangzhou Institute of Respiratory Health, The First Affiliated Hospital of Guangzhou Medical University, Guangzhou, China

**Keywords:** polycystic ovary syndrome (PCO), sleep disturbances, relationship, meta-analysis', risk

## Abstract

**Objective:** A meta-analysis is used to explore the relationship between polycystic ovary syndrome (PCOS) and the risk of Sleep disturbances.

**Method:** Cochrane Library, PubMed, Embase, and Web of Science databases are searched by computer from their establishment to 1 May 2022. Review Manager 5.4 software is used for the meta-analysis.

**Results:** A total of nine articles are included, with 1,107 subjects. The results show that PCOS is positively associated with the risk of Sleep disturbances. Comparing with the “PCOS group” (experimental group) with the “NON-PCOS group” (control group), the incidence of Sleep disturbances is higher (OR = 11.24, 95% CI: 2.00–63.10, Z = 2.75, *p* = 0.006); the Pittsburgh Sleep Quality Index (PSQI) scores of the PCOS group is higher than that of the NON-PCOS group (MD = 0.78, 95% CI: 0.32–1.25, Z = 3.30, *p* = 0.001); the Epworth Sleepiness Scale (ESS) scores of the PCOS group is higher than that of the NON-PCOS group (MD = 2.49, 95% CI: 0.80–4.18, Z = 2.88, *p* = 0.004); Apnea hypopnea index (AHIs) in the PCOS group are higher than those in the NON-PCOS group (MD = 2.68, 95% CI: 1.07–4.28, Z = 3.27, *p* = 0.001); the sleep efficiency of the PCOS group is lower than that of the NON-PCOS group (MD = -5.16, 95% CI: 9.39–-0.93, Z = 2.39, *p* = 0.02); the sleep onset latency of the PCOS group is higher than that of the NON-PCOS group (MD = 2.45, 95% CI: 1.40–3.50, Z = 4.57, *p* < 0.001); and the Rapid Eyes Movement (REM) sleep in the PCOS group is higher than that in the NON-PCOS group (MD = 17.19, 95% CI: 11.62–55.76, Z = 6.05, *p* < 0.001). The studies included in each analysis have publication biases of different sizes. After subgroup analysis and sensitivity analysis, the heterogeneity of each study in the meta-analysis is reduced, the bias is reduced accordingly, and the stability of the results can be maintained.

**Conclusion:** PCOS is positively associated with the risk of Sleep disturbances. In order to reduce such risk, attention should be paid to the role of PCOS management, and PCOS prevention and treatment should be actively carried out.

## 1 Introduction

Polycystic ovary syndrome (PCOS) is the most common endocrine disorder in women of reproductive age (4–12%) (Azziz et al., 2004), while its prevalence depends on the choice of diagnostic criteria. A community-based prevalence study using the Rotterdam criteria found that approximately 18% of women had PCOS, 70% of whom had not been diagnosed previously (Teede et al., 2010). PCOS usually occurs during puberty with irregular menstrual cycles, signs of hyperandrogenism such as acne and hirsutism, and insulin resistance (Azziz, 2006). PCOS was discovered in the 1930s, but the understanding of its relationship with Sleep disturbances was relatively late. Most studies on this topic were published after 2001, and the research content is relatively small, with some variations in results. Recent studies have linked PCOS to Sleep disturbances, with a prospective case-control study estimating that women with PCOS have a 30-fold higher prevalence of Sleep disturbances than women in the general population (Nandalike et al., 2011). Studies have confirmed that persistent lack of sleep can lead to decreased thinking ability and memory, decreased vigilance and judgment, low immunity, endocrine disorders, anxiety, and irritability, and ultimately lead to the occurrence of diseases such as high blood pressure, cardiovascular and cerebrovascular diseases, and affective psychosis, aggravate the severity of age-related chronic diseases, and increase the risk factors of periodic coronary heart disease in middle-aged and elderly women (Tsay et al., 2003). Sleep itself is an important regulator of endocrine function, and the endocrine system also plays an important role in regulating the sleep-wake cycle (Calandra-Buonaura et al., 2016). There may be a complex relationship between PCOS as an endocrine disorder and sleep. Therefore, this study conducted a meta-analysis of the risk relationship between Sleep disturbances and PCOS in order to provide medical evidence for an exploration of the etiology of PCOS and its preventive treatment.

## 2 Materials and methods

### 2.1 Retrieval strategy

According to the meta-analysis of Observational Studies in Epidemiology (MOOSE) statement and standard of preferred reporting items for Systematic Reviews and meta-analyses (PRISMA), and the PRISMA checklist item is depicted in Supplementary File S1. Cochrane Library, PubMed, Embase, and Web of Science databases were searched by computer from their establishment to 1 May 2022. English search terms included “polycystic ovary syndrome”, “PCOS”, “Stein-leventhal Syndrome”, “Sclerocystic Ovarian Degeneration”, “Ovary Syndrome, Polycystic”, “Syndrome, Polycystic Ovary”, “Stein-Leventhal Syndrome”, “Stein Leventhal Syndrome”, “Syndrome, Stein-Leventhal”, “Ovarian Degeneration, Sclerocystic”, “Sclerocystic Ovary Syndrome”, “Polycystic Ovarian Syndrome”, “Sclerocystic Ovaries”, “Ovary, Sclerocystic","Sclerocystic Ovary”, Sleep Wake Disorders","Disorder, Sleep Wake”, “sleep disturbances”, “Disorder, Sleep Wake”, “Disorders, Sleep Wake”, “Sleep Wake Disorder”, “Wake Disorder, Sleep”, “Wake Disorders, Sleep Disorders”, “Disorder, Sleep”, “Disorders, Sleep”, “Sleep Disorder”, “Short Sleeper Syndrome”, “Short Sleeper Syndromes”, “Sleeper Syndrome, Short”, “Sleeper Syndromes, Short”, “Syndrome, Short Sleeper”, “Syndromes, Short Sleeper”, “Short Sleep Phenotype”, “Phenotype, Short Sleep”, “Phenotypes, Short Sleep”, “Short Sleep Phenotypes”, “Sleep Phenotypes, Short”, “Sleep”. The details of the search process for each database are listed in Supplementary File S2. The type of research design was not limited, and the language was limited to English.

### 2.2 Literature inclusion and exclusion criteria

Inclusion criteria: ① the subjects of the study were patients with a clinical diagnosis of PCOS in the case group, and healthy women with a normal menstrual cycle or female patients who came to the hospital for other reasons in the control group; ② the types of studies were cohort studies, case-control studies, and cross-sectional studies which were compared with the control group; ③ articles in which the subjects were diagnosed as having PCOS by the criteria of National Institutes of Health (NIH; 1990) (Zawadzki et al., 1992), and Rotterdam criteria (AE-PCOS; 2003) (Rotterdam ESHRE/ASRM-Sponsored PCOS Consensus Workshop Group, 2004), and Sleep disturbances were diagnosed and investigated by polysomnography, actiwatch, and sleep questionnaires.

Exclusion criteria: ① languages other than English; ② replicated publications (if studies involving the same population were published repeatedly, the latest published studies or those with the larger sample size were selected); ③ studies without a control group; ④ studies for which the effect sizes could not be extracted or calculated; ⑤ studies with little research information or incomplete data and inconsistent outcome indicators; ⑥ studies on the use of contraceptives, metformin and other drugs that affect PCOS; ⑦ studies in which the population is accompanied by hypertension, diabetes, atypical adrenal 21-hydroxylase deficiency, androgen-secreting tumor, Cushing’s syndrome, and any other disease that may cause Sleep disturbances; ⑧ the experimental group and control group had significant body mass index (BMI) differences (differences greater than 10 kg/m^2^ were excluded) which were likely to cause bias.

### 2.3 Literature screening, quality assessment, and data extraction

Two researchers independently searched, extracted and screened the literature, then checked each other’s work, and provided articles with differences to a third researcher to decide whether they should be included. The methodological quality of the included literature was assessed. The evaluation was performed using the “Risk of Bias Assessment” tool recommended by the Cochrane Collaboration, which is divided into three levels: low risk, unclear, and high risk. The content is: whether it is randomly assigned; whether to perform allocation concealment; whether to use blinding; whether the outcome data is complete; whether the research results are selectively reported; and whether there is other risk of bias. The extracted data included the first author, study area, publication time, sample size, age, BMI, prevalence of Sleep disturbances, AHIs, sleep efficiency, rapid eye movement (REM) sleep, sleep onset latency, Pittsburgh Sleep Quality Index (PSQI), Epworth Sleepiness Scale (ESS), outcome measures, and adjustment for confounders. After data extraction, the two datasets were checked, and inconsistent data was extracted again. After checking, the data was analyzed.

### 2.4 Ending and exposure

The PCOS of the research subjects had to meet the NIH or Rotterdam diagnostic criteria, and Sleep disturbances were diagnosed by polysomnography, actiwatch, and sleep questionnaires. The prevalence of Sleep disturbances (including sleep apnea or obstructive sleep apnea) in the PCOS group and control group, AHIs, sleep efficiency, REM sleep, sleep onset latency, PSQI, and ESS were used as outcome indicators. The incidence of Sleep disturbances between the PCOS group and control group was different, indicating a correlation between PCOS and Sleep disturbances; comparing with the PCOS group and control group, the differences in levels of AHIs, sleep efficiency, REM sleep, sleep onset latency, PSQI, and ESS were included, illustrating the effects of PCOS on Sleep disturbances.

### 2.5 Statistical methods

Statistical analysis was performed using Review Manager 5.4 software. Quantitative data was expressed as mean ± standard deviation (x ± s), MD and OR values were used for effect evaluation, and 95% CI was calculated. The I^2^ statistical value test and Q test were used to analyze the heterogeneity of the studies. If the heterogeneity among the studies was not statistically significant (I^2^ ≤ 50% and *p* ≥ 0.1), a fixed effects model was used; and if there was heterogeneity (I^2^ > 50% and *p* < 0.1), a random effects model was used. Sensitivity analysis was used to judge the stability and reliability of the combined results. *p* < 0.05 was considered statistically significant.

## 3 Results

### 3.1 Literature screening results

**FIGURE 1:**
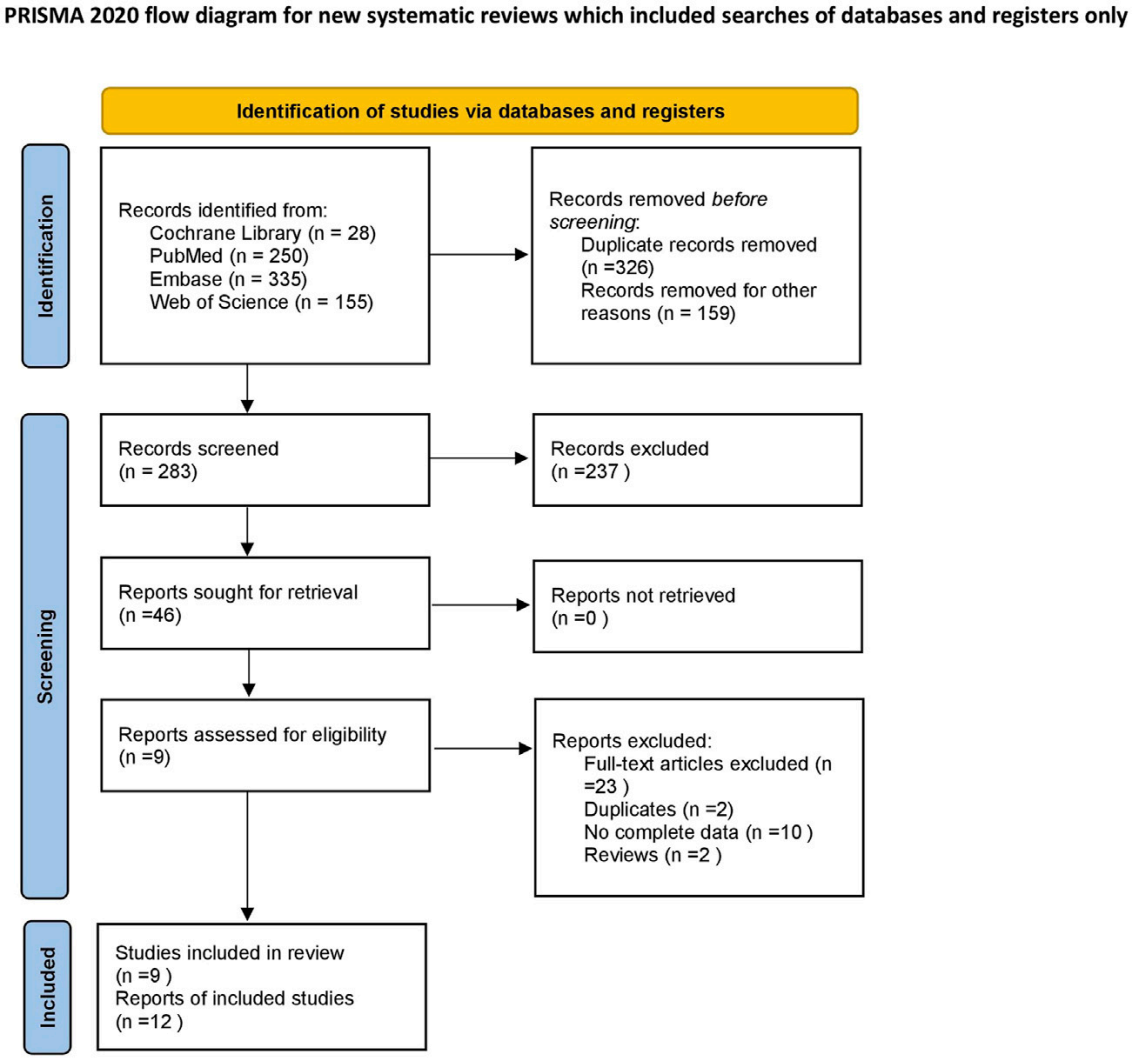
Flow chart of literature screening.

A total of 768 articles were retrieved, of which 442 papers were obtained after deduplication, and 237 papers were excluded by reading the titles and abstracts. After reading the full texts, nine papers (Fogel et al., 2001; Tasali et al., 2008a; Yang et al., 2009; de Sousa et al., 2010; de Sousa et al., 2011; Shreeve et al., 2013; Suri et al., 2016; Su et al., 2017; Azizi et al., 2020) were finally included (Figure 1), with a total of 1,107 research subjects. The study populations were from United States; China; Taiwan, China; Germany; Britain; India; and Iran. The basic characteristics of the literature included in the study are shown in Table 1.

**TABLE 1:** Basic features of the included studies.

Study	Country	Ages (Years)	Sample size n (T/C)	Criteria for PCOS	Criteria for SDB	BMI (kg/m2)	Research object characteristics
Fogel, 2001 (Fogel et al., 2001)	United States	T: 31.1 ± 1.3, C: 32.3 ± 1.3	36 (18/18)	NIH	Polysomnography/Sleep questionnaire	T: 36.96 ± 1.3, C: 36.96 ± 1.4	no concomitant disease
Tasali 2008 (Tasali et al., 2008a)	United States	18–40	73 (52/21)	NIH	Polysomnography	>25 kg/m2	no concomitant disease
Yang 2009 (Yang et al., 2009)	Taiwan, China	T: 29.1 ± 1.43	28 (18/10)	Rotterdam	Polysomnography/Sleep questionnaire	T: 21.7 ± 0.57	non-obese
		C: 31.6 ± 3.87				C: 20.9 ± 0.58	no concomitant disease
Sousa 2010 (de Sousa et al., 2010)	Germany	T: 15.2 ± 1.3	33 (22/11)	NIH	Polysomnography	T: 31.7 ± 6.2	no concomitant disease
		C: 15.0 ± 1.0				C: 34.8 ± 8.7	
Sousa 2011 (de Sousa et al., 2011)	Germany	T: 15.0 ± 1.0	50 (31/19)	NIH	Polysomnography	T: 32.7 ± 6.2	no concomitant disease
		C: 15.2 ± 1.1				C: 32.4 ± 4.0	
Shreeve 2013 (Shreeve et al., 2013)	Britain	T: 29.8 ± 3.7	52 (26/26)	Rotterdam	actiwatch/Sleep questionnaire	T: 29.3 ± 8.2	no concomitant disease
		C: 26.3 ± 5.6				C: 24.6 ± 3.3	
Suri 2016 (Suri et al., 2016)	India	T: 27.9 ± 6.44	150 (50/100)	Rotterdam	Polysomnography/Sleep questionnaire	T: 28.0 ± 4.01	no concomitant disease
		C: 28.3 ± 6.05				C: 25.3 ± 2.93	
Su 2017 (Su et al., 2017)	China	T: 29.03 ± 3.26	285 (129/156)	Rotterdam	Sleep questionnaire	Unlimited	no concomitant disease
		C: 31.72 ± 3.86					
Kutenaee 2019 (Azizi et al., 2020)	Iran	T: 27.86 ± 5.84	400 (201/199)	Rotterdam	Sleep questionnaire	T: 22.73 ± 9.62	no concomitant disease
		C: 28.06 ± 6.51				C: 23.95 ± 4.96	

Abbreviations: PCOS, polycystic ovary syndrome; SDB, sleep-disordered breathing; BMI, body mass index; NIH, national institutes of health.

### 3.2 Quality evaluation of included studies

The Newcastle–Ottawa (NOS) scale was used to evaluate the quality of the included observational studies. The specific evaluation is shown in Table 2. The lowest overall evaluation was 6★ and the highest was 8★, all of which were of high quality. All studies had a low to moderate risk of bias and no studies were excluded due to poor quality (<5★).2.3 Statistical Analysis Results.

**TABLE 2:** Newcastle–Ottawa Scale of the included studies.

Study	Year	Selection	Comparablity	Exposure	Quality scoress
Fogel	2001	★★★★	★★	★★	8
Tasali	2008	★★★★	★★	★	7
Yang	2009	★★★★	★★	★★	8
Sousa	2010	★★★★	★	★★	7
Sousa	2011	★★★★	★★	★	7
Shreeve	2013	★★★	★	★★★	7
Suri	2016	★★★	★	★★	6
Su	2017	★★★	★★	★★★	8
Kutenaee	2019	★★★	★★	★★	7

#### 3.3.1 Association between PCOS and Incidence of Sleep disturbances

**FIGURE 2:**
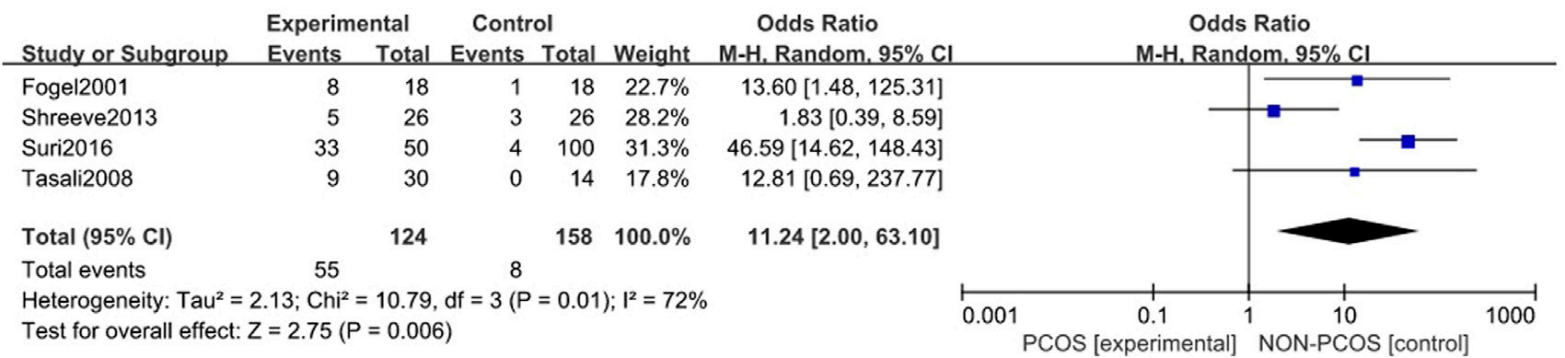
Forest plot of the incidence of sleep disturbance in the PCOS group and the control group (before heterogeneity was excluded).

**FIGURE 3:**
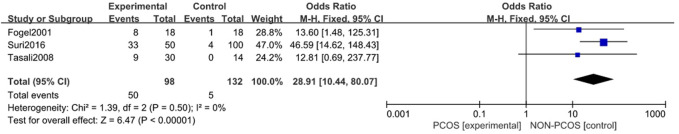
Forest plot of incidence of sleep disturbance in PCOS group and control group (after removing heterogeneity studies).

A total of four studies were included (Fogel et al., 2001; Tasali et al., 2008a; Shreeve et al., 2013; Suri et al., 2016), all of which were used in the analysis. There was moderate heterogeneity among the included studies (*p* = 0.01, I^2^ = 72%), so a random effects model was used for analysis (Figure 2). The results showed that the incidence of Sleep disturbances in the PCOS group was higher than in the control group (OR = 11.24, 95% CI: 2.00–63.10, Z = 2.75, *p* = 0.006), and the results were statistically significant. In order to reduce the clinical heterogeneity of the research subjects, the sensitivity analysis of the incidence of Sleep disturbances showed that there was no heterogeneity after excluding one study: Shreeve 2013 (Shreeve et al., 2013) (*p* = 0.5, I^2^ = 0%). Furthermore, the fixed effects model analysis showed (Figure 3) that the incidence of Sleep disturbances in the PCOS group was higher than in the control group (OR = 28.91, 95% CI: 10.44–80.07, Z = 6.47, *p* < 0.001), further verifying that Sleep disturbances in the PCOS group had a higher prevalence and statistical significance. Looking back at the original text, it was found that the sleep disorder assessment method of Shreeve 2013 (Shreeve et al., 2013) was actiwatch, while the assessment method of the other studies was polysomnography. Considering that the generation of heterogeneity may be related to the method of sleep disorder assessment, there is a certain degree of heterogeneity, suggesting that future research can specifically explore the effects of sleep disorder assessment methods on the study population.

#### 3.3.2 Correlation between PCOS and PSQI scores

**FIGURE 4:**
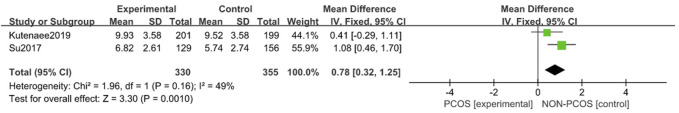
Forest plot of PSQI scores of sleep disturbance in PCOS group and control group.

A total of two studies were included (de Sousa et al., 2011; Shreeve et al., 2013), both of which were used in the analysis (Figure 4). The heterogeneity among the included studies was mild (*p* = 0.16, I^2^ = 49%), so a fixed effects model was used for analysis. To enter the forest plot of the PSQI scores of Sleep disturbances in the control group. The results showed that the PSQI scores of PCOS group was higher than that of control group (MD = 0.78,95% CI: 0.32–1.25, z = 3.30, *p* = 0.001); In general, the prevalence of sleep disorders in PCOS group was higher, which was statistically significant.

#### 3.3.3 Correlation of PCOS and ESS scoress

**FIGURE 5:**
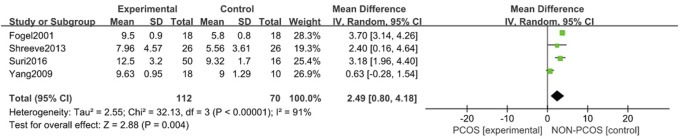
Forest plot of ESS scoress in PCOS and control groups (before heterogeneity was excluded).

**FIGURE 6:**
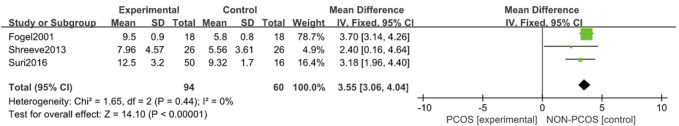
Forest plot of ESS scoress in PCOS group and control group (after removing heterogeneity studies).

A total of four studies were included (Fogel et al., 2001; Yang et al., 2009; Shreeve et al., 2013; Suri et al., 2016), all of which were used in the analysis. There was large heterogeneity among the included studies (*p* < 0.001, I^2^ = 91%), so a random effects model was used for analysis (Figure 5). The results showed that the ESS scores in the PCOS group was higher than in the control group (MD = 2.49, 95% CI: 0.80–4.18, Z = 2.88, *p* = 0.004), with statistical significance. In order to reduce the clinical heterogeneity of the research subjects, the sensitivity analysis for the ESS scoress showed that there was no heterogeneity after excluding one study, Yang 2009 (Yang et al., 2009) (*p* = 0.44, I^2^ = 0%); further fixed effects model analysis was performed, showing (Figure 6) that the ESS scoress of the PCOS group was higher than that of the control group (MD = 3.55, 95% CI: 3.06–4.04, Z = 14.1, *p* < 0.001), with statistical significance. Looking back at the original text, it was found that the mean BMI of the study population in Yang 2009 (Yang et al., 2009) was lower than 24 kg/m^2^, while the mean BMI of the other study populations was higher than 28 kg/m^2^, so it was considered that the generation of heterogeneity may be related to the BMI value of the study population. Therefore, there was a certain heterogeneity, suggesting that the effects of BMI on PCOS and Sleep disturbances in the study population should be explored in future studies.

#### 3.3.4 Correlation of PCOS with AHIs

**FIGURE 7:**
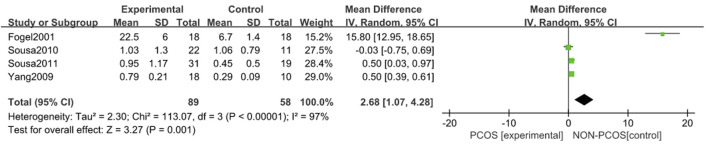
Forest plot of AHIs in PCOS group and control group.

A total of four studies were included (Fogel et al., 2001; Tasali et al., 2008a; Suri et al., 2016; Su et al., 2017), all of which were used in the analysis (Figure 7). There was great heterogeneity among the included studies (*p* < 0.001, I^2^ = 97%), so a random effects model was used for analysis. The results showed that AHIs in the PCOS group were higher than those in the control group (MD = 2.68, 95% CI: 1.07–4.28, Z = 3.27, *p* = 0.001), with statistical significance.

#### 3.3.5 Correlation between PCOS and sleep efficiency

**FIGURE 8:**
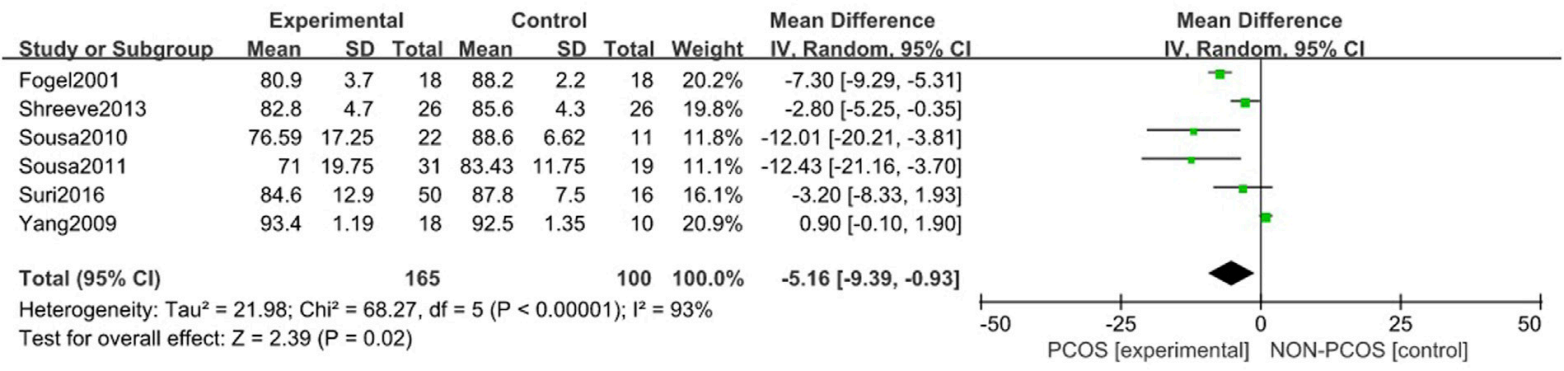
Forest plot of sleep efficiency correlation between PCOS group and control group.

**FIGURE 9:**
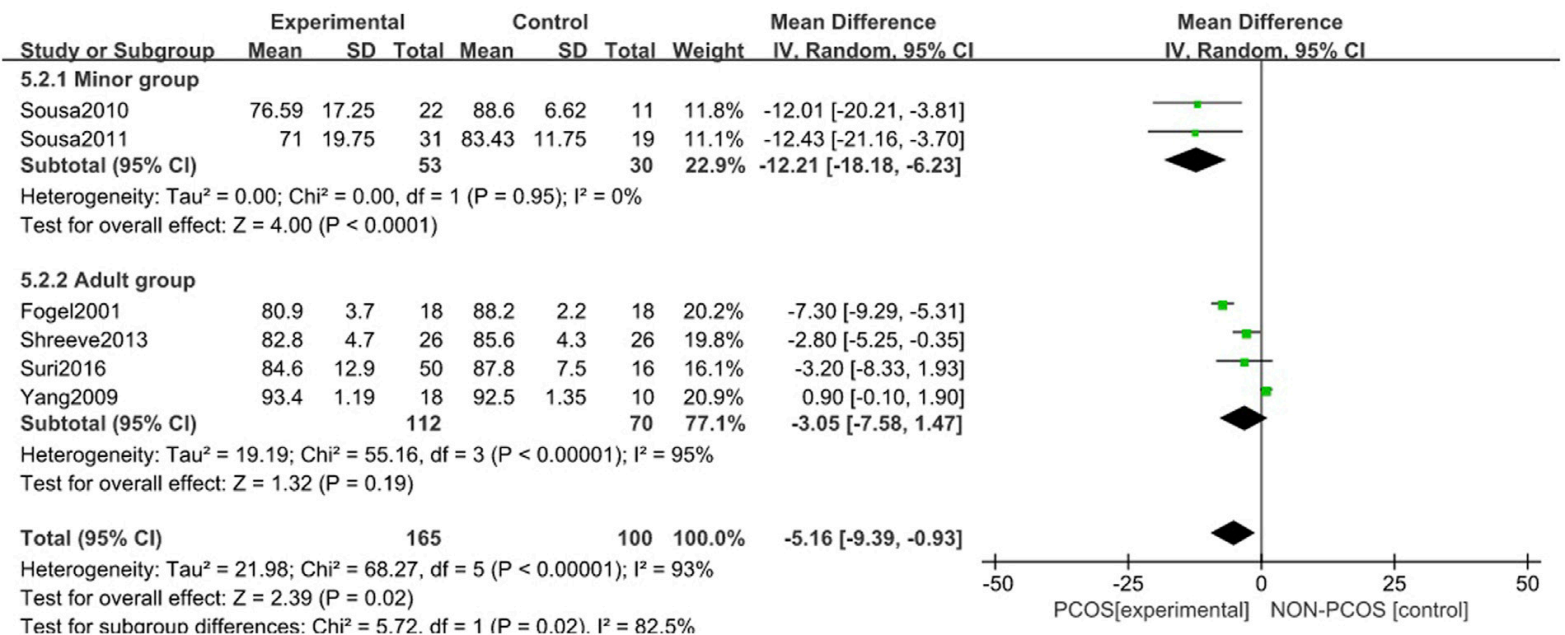
Forest plot of sleep efficiency correlation between PCOS group and control group (age subgroup).

A total of six studies were included (Fogel et al., 2001; Yang et al., 2009; de Sousa et al., 2010; Suri et al., 2016; Su et al., 2017; Azizi et al., 2020), all of which were used in the analysis (Figure 8). There was great heterogeneity among the included studies (*p* < 0.001, I^2^ = 93%), so a random effects model was used for analysis. The results showed that the sleep efficiency of the PCOS group was lower than that of the control group (MD = -5.16, 95% CI: 9.39–-0.93, Z = 2.39, *p* = 0.02), which was statistically significant, but the included studies were heterogeneous. To further verify the relationship between PCOS and sleep efficiency, and further reduce the clinical heterogeneity of the study subjects, a subgroup analysis of the study population was performed (Figure 9) in which the heterogeneity of the minor group was (*p* = 0.95, I^2^ = 0%), and the heterogeneity of the adult group was (*p* < 0.001, I^2^ = 95%). These results showed that compared with the control group, the PCOS group was divided into minors (MD = -12.21, 95% CI: -18.18–-6.23, Z = 4, *p* < 0.001) and adults (MD = - 0.08, 95% CI: -0.19–0.02, Z = 1.51, *p* = 0.13), there was no heterogeneity in the subgroup study of minors, the sleep efficiency of the PCOS population in the minor subgroup was lower, and the difference was statistically significant. However, there was large heterogeneity in the adult subgroup study, and the sleep efficiency of the PCOS population in the adult subgroup was low, but the difference was not statistically significant. Considering that the generation of heterogeneity may be related to age, the influencing factor of age needs to be further confirmed by future large-sample, multi-center clinical studies.

#### 3.3.6 Correlation between PCOS and Sleep Onset Latency

**FIGURE 10:**
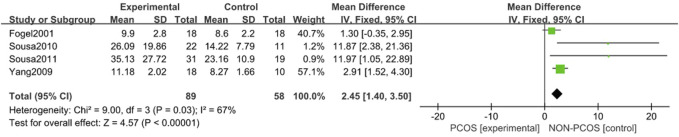
Forest plot of correlation between sleep onset latency in PCOS group and control group.

A total of four studies were included (Fogel et al., 2001; Suri et al., 2016; Su et al., 2017; Azizi et al., 2020), all of which were used in the analysis (Figure 10). There was moderate heterogeneity among the included studies (*p* = 0.03, I^2^ = 67%), so a random effects model was used. The results showed that the sleep onset latency in the PCOS group was longer than that in the control group (MD = 2.45, 95% CI: 1.40–3.50, Z = 4.57, *p* < 0.001), which was statistically significant.

#### 3.3.7 Correlation between PCOS and REM sleep

**FIGURE 11:**
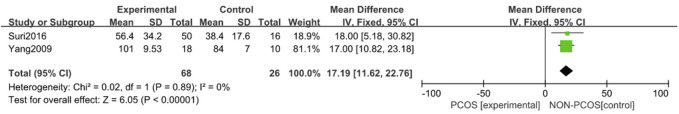
Forest plot of REM sleep correlation between PCOS group and control group.

A total of two studies were included (de Sousa et al., 2010; Suri et al., 2016), both of which were included in the analysis (Figure 11). There was no heterogeneity among the included studies (*p* = 0.89, I^2^ = 0%), so a fixed effects model was used for analysis. The results showed that the REM sleep in the PCOS group was higher than that in the control group (MD = 17.19, 95% CI: 11.62–55.76, Z = 6.05, *p* < 0.001); overall, the REM sleep in the PCOS group was higher, with statistical significance.

### 3.4 Sensitivity analysis

In this study, sensitivity analysis was conducted for the results with high heterogeneity and statistical significance. In the sensitivity analysis of the incidence of sleep disorders in the PCOS group and the control group, there was no significant change in the combined results after excluding any literature, and the results were stable; After the Shreeve 2013 (Shreeve et al., 2013) study was excluded, there was no heterogeneity among the studies. Tracing back to the original text, it was found that the sleep disorder evaluation method of this study was actiwatch, while the evaluation method of other studies was polysomnography. Considering that the generation of heterogeneity may be related to the sleep disorder evaluation method, there was some heterogeneity, suggesting that the effect of sleep disorder evaluation methods on the study population can be explored in future studies. In the sensitivity analysis of the ESS score in the PCOS group and the control group, there was no significant change in the combined results after excluding any literature, and the results were stable; After the Yang 2009 (Yang et al., 2009) study was excluded, the heterogeneity of the study decreased significantly. It was found that the average BMI of the study population was lower than 24 kg/m^2^, while the average BMI of other study populations was higher than 28 kg/m^2^. Considering that the generation of heterogeneity may be related to the BMI value of the study population, there was a certain heterogeneity, suggesting that the effect of BMI of the study population on PCOS and sleep disorders can be explored in future studies. In the sensitivity analysis of the AHI in the PCOS group and the control group, there was no significant change in the combined results after excluding any literature, and the results were stable. After the fogel 2001 (Fogel et al., 2001) study was excluded, the heterogeneity of the study decreased significantly. In the sensitivity analysis of thesleep onset latency in the PCOS group and the control group, there was no significant change in the combined results and inter study heterogeneity after excluding any literature.

### 3.5 Publication bias

The presence of publication bias was assessed using Egger’s method. There were four studies on the incidence of Sleep disturbances in the PCOS group and control group. The result of Egger’s method was *p* = 0.664 > 0.05, indicating no significant publication bias. There were four studies on the ESS score in the PCOS group and control group. The result of Egger’s method was *p* = 0.596 > 0.05, indicating no significant publication bias. There were four studies on the AHI in the PCOS group and control group. The result of Egger’s method was *p* = 0.427 > 0.05, indicating no significant publication bias. There were six studies on the Sleep Efficiency in the PCOS group and control group. The result of Egger’s method was *p* = 0.124 > 0.05, indicating no significant publication bias. There were four studies on the Sleep Onset Latency in the PCOS group and control group. The result of Egger’s method was *p* = 0.183 > 0.05, indicating no significant publication bias.

## 4 Discussion

PCOS is a complex endocrine disorder that affects reproductive, metabolic, and mental health in women. Clinically-based studies have shown that Sleep disturbances, including obstructive sleep apnea and excessive daytime sleepiness, occur more frequently in women with PCOS compared with control groups of women without PCOS (Fernandez et al., 2018). Sleep is an important part of normal physiology, and Sleep disturbances are common in contemporary society. Abnormal sleep is associated with health conditions and comorbidities such as obesity, hypertension, diabetes, depression, and poor quality of life. Little is known about the relationship between female sleepiness and PCOS, and several limited studies investigating sleep in women of reproductive age suggest that PCOS is associated with Sleep disturbances (Ehrmann, 2005). Women with PCOS also have an increased incidence of depression (Cooney and Dokras, 2018) and metabolic dysfunction (Dunaif et al., 1989), both of which are associated with Sleep disturbances.

Sleep disturbances and disorders can affect many aspects of daytime mood, cognition, and psychomotor function, so Sleep disturbances is a serious health problem. Recognized Sleep disturbances include features of sleep deprivation and insomnia (difficulty falling asleep, difficulty maintaining sleep, waking up too early, or unrefreshing sleep), which may be problematic for the individual, even below the clinically defined 1-month duration time (Moran et al., 2015). Hallmark sequelae include daytime fatigue, lethargy, and irritability. Sleep disturbances can occur in the absence of OSA (in which breathing is disturbed during sleep). Notably, women’s subjective experience of Sleep disturbances differs from men’s, with insomnia and subsequent depression more characteristic than men’s typical snoring, apnea, and daytime sleepiness (Redline et al., 1994; Pillar and Lavie, 1998). At present, there are various ways to evaluate sleep quality, including objective monitoring methods such as polysomnography and various subjective questionnaires such as the PSQI (Buysse et al., 1989), ESS (Johns, 1991), and Leeds sleep evaluation questionnaire (LSEQ) (Skrobik et al., 2018).

A total of nine studies were included in this meta-analysis. The sleep quality of patients was further explored through the analysis of multiple indicators. The results showed that PCOS is closely related to Sleep disturbances, and may increase the risk of Sleep disturbances; among them, the incidence of Sleep disturbances is higher in PCOS patients; PSQI scoress, ESS scoress, AHIs, sleep onset latency, and REM sleep were higher in PCOS patients than those in the control group, the sleep efficiency of PCOS patients was lower than that of the control group, and PCOS patients all had more serious sleep problems.

Specifically, the results of the meta-analysis of the relation between PCOS and Sleep disturbances risk showed that the incidence of Sleep disturbances in PCOS patients was high; after excluding one study, Shreeve 2013 (Shreeve et al., 2013), there was no heterogeneity and no obvious research bias, indicating that this study was the source of the heterogeneity and bias, and that the research conclusions of the other articles were more reliable. However, only four studies were included, so analyzing a larger number of studies can help to further stabilize the research results. When PCOS patients were analyzed and evaluated in terms of PSQI scores, ESS scores, AHIs, sleep onset latency, REM sleep, etc., the degree of Sleep disturbances in PCOS patients was more serious. Prolonged sleep efficiency decreased and REM sleep increased, but excluding any studies had no significant effect on the combined effect size of the remaining literature, confirming that the results were relatively stable, but the sleep efficiency, PSQI, and ESS studies had no statistical significance after deleting any studies, and the number of included studies was not statistically significant. Thus, the amount of data was insufficient, and further confirmation by large-sample, multi-center clinical studies is required.

The pathophysiological mechanism leading to the high incidence of Sleep disturbances in PCOS has not yet been determined, and some studies simply attribute it to the presence of excessive BMI or obesity in women with PCOS, but this is only part of the reason for their sleep problems, as this association persisted after adjusting for the index (de Sousa et al., 2010), and PCOS patients with normal BMI also experience sleep problems. SDB is an independent risk factor for metabolic dysfunction in women with PCOS (Vgontzas et al., 2001). This meta-analysis also showed that sleep parameters were abnormal in both the overweight PCOS population and normal weight PCOS population. At present, it is believed that there are several pathways in the relation of PCOS to Sleep disturbances, and they may be bidirectional: (1) Insulin resistance is an important pathological feature of PCOS, and studies have shown that Sleep disturbances can exacerbate insulin resistance (Rao et al., 2015), while insulin increases the sympathetic nervous system; this, this may in turn affect sleep architecture, increasing the risk of sleep-disordered breathing and daytime sleepiness (Greco and Spallone, 2015). (2) Women with PCOS have elevated hypothalamic-pituitary-adrenal axis reactivity (Benson et al., 2009), which is associated with Sleep disturbances (Vgontzas, 2008); the circadian sleep-wake cycle also involves peripheral regulation by neurotransmitters and neuromodulators, including melatonin and cytokines. (3) Women with PCOS have an altered cytokine profile (Xiong et al., 2011) and increased diurnal urine levels of the melatonin metabolite 6-sulfamoyloxymelatonin (Luboshitzky et al., 2001), which in turn can affect sleep. (4) Many women with PCOS have bad living habits such as smoking, alcoholism, and lack of physical activity, which can lead to Sleep disturbances (Thakkar et al., 2015; Zomers et al., 2017). (5) The disorders of the stress system in women with PCOS leads to the disorders of the sleep cycle (Vgontzas et al., 2001). (6) Alterations in body fat composition due to excess androgen levels and/or the effects of metabolic syndrome (Tasali et al., 2008b) have been previously associated with an increased risk of Sleep disturbances in patients without PCOS.

Limitations of this study: ① The number of included studies on the correlation between PCOS and Sleep disturbances was small, and the results were not stable, so further research is required to stabilize the research results; ② the included studies were case-control studies carried out to demonstrate the weak strength of the causal relationship, and their overall evidence-based medicine level was low; ③ the diagnostic methods of PCOS and sleep disorder assessment methods in each study were slightly different, and the study populations were from different age groups, which may have caused a certain level of heterogeneity in the results; ④ Sleep disturbances are more common in obese people (Koren and Taveras, 2018), and the populations of the studies included in our meta-analysis mainly consisted of obese patients and few patients with normal BMI, so there may be selection bias. Therefore, the conclusions of this meta-analysis should be interpreted with caution.

## 5 Conclusion

The results of this study suggest that the incidence of Sleep disturbances in patients with PCOS is high. PSQI scores, ESS scores, AHIs, sleep efficiency, sleep onset latency, and REM sleep may be related to PCOS. In order to understand the incidence and prevention of Sleep disturbances in patients with PCOS Treatment provides evidence-based medical evidence. Effective PCOS prevention and treatment can effectively reduce the risk of Sleep disturbances. Limited by the quantity and quality of included studies, the above conclusions need to be further confirmed by more large-sample, multi-center clinical studies.

## Data Availability

The raw data supporting the conclusions of this article will be made available by the authors, without undue reservation.
