# Supplementary material for: A meta-analysis of the relationship between polycystic ovary syndrome and sleep disturbances risk
Source: Front Physiol. 2022 Sep 29;13:957112. doi: 10.3389/fphys.2022.957112 (PMC9558285; doi:10.3389/fphys.2022.957112)
Supplement: Supplementary file 1 [file Table1.docx]

Supplementary materials

Supplementary Table 1.**Full search strategy for each of the electronic databases queried.**

|  |
| --- |
| **PubMed (including Web of Science)** Coverage: Date of inception 1940s – May 1st, 2022  Vendor/platform: National Library of Medicine Hits: 250 |
| PubMed was searched with appropriate Medical Subject Headings (MeSH) incorporated into hedges. Filters were set for Humans:  (((("Sleep Wake Disorders"[Mesh]) OR (((((((((((((((((((((Disorder, Sleep Wake) OR (sleep disturbances)) OR (Disorder, Sleep Wake)) OR (Disorders, Sleep Wake)) OR (Sleep Wake Disorder)) OR (Wake Disorder, Sleep)) OR (Wake Disorders, Sleep Sleep Disorders)) OR (Disorder, Sleep)) OR (Disorders, Sleep)) OR (Sleep Disorder)) OR (Short Sleeper Syndrome)) OR (Short Sleeper Syndromes)) OR (Sleeper Syndrome, Short)) OR (Sleeper Syndromes, Short)) OR (Syndrome, Short Sleeper)) OR (Syndromes, Short Sleeper)) OR (Short Sleep Phenotype)) OR (Phenotype, Short Sleep)) OR (Phenotypes, Short Sleep)) OR (Short Sleep Phenotypes)) OR (Sleep Phenotypes, Short))) OR (sleep)) AND (("Polycystic Ovary Syndrome"[Mesh]) OR ((((((((((((((((PCOS) OR (stein -leventhal Syndrome)) OR (Sclerocystic Ovarian Degeneration)) OR (Ovary Syndrome, Polycystic)) OR (Syndrome, Polycystic Ovary)) OR (Stein-Leventhal Syndrome)) OR (Stein Leventhal Syndrome)) OR (Syndrome, Stein-Leventhal)) OR (Ovarian Degeneration, Sclerocystic)) OR (Sclerocystic Ovary Syndrome)) OR (Polycystic Ovarian Syndrome)) OR (Ovarian Syndrome, Polycystic)) OR (Polycystic Ovary Syndrome 1)) OR (Sclerocystic Ovaries)) OR (Ovary, Sclerocystic)) OR (Sclerocystic Ovary)))) |
|  |
| **EMBASE**  Coverage: Date of inception 1960 – May 1st, 2022  Vendor/platform: Elsevier SciVerse Hits: 355 |
| **Search #1:**  **Line 1**:[Sleep Wake Disorders] OR [Disorder, Sleep Wake] OR [sleep disturbances] OR [Disorder, Sleep Wake] OR [Disorders, Sleep Wake] OR [Sleep Wake Disorder] OR [Wake Disorder, Sleep] OR [Wake Disorders, Sleep Sleep Disorders] OR [Disorder, Sleep] OR [Disorders, Sleep] OR [Sleep Disorder] OR [Short Sleeper Syndrome] OR [Short Sleeper Syndromes] OR [Sleeper Syndrome, Short] OR [Sleeper Syndromes, Short] OR [Syndrome, Short Sleeper] OR [Syndromes, Short Sleeper] OR [Short Sleep Phenotype] OR [Phenotype, Short Sleep] OR [Phenotypes, Short Sleep] OR [Short Sleep Phenotypes] OR [Sleep Phenotypes, Short] OR [Sleep]  **Search #2:**  **Line 1**: "Polycystic Ovary Syndrome" OR "PCOS" OR "stein -leventhal Syndrome" OR "Sclerocystic Ovarian Degeneration" OR "Ovary Syndrome, Polycystic" OR "Syndrome, Polycystic Ovary" OR "Stein-Leventhal Syndrome" OR "Stein Leventhal Syndrome" OR "Syndrome, Stein-Leventhal" OR "Ovarian Degeneration, Sclerocystic" OR "Sclerocystic Ovary Syndrome" OR "Polycystic Ovarian Syndrome" OR "Ovarian Syndrome, Polycystic" OR "Polycystic Ovary Syndrome 1" OR "Sclerocystic Ovaries" OR "Ovary, Sclerocystic" OR "Sclerocystic Ovary"  **Then under search history: combined search #1 and #2** |
|  |
| **Cochrane Library** Coverage: Date of inception 1999 – May 1st, 2022  Vendor/Platform: Wiley Online Library Hits: 28 |
| **Line 1**:"Sleep Wake Disorders" OR "Disorder, Sleep Wake" OR "sleep disturbances" OR "Disorder, Sleep Wake" OR "Disorders, Sleep Wake" OR "Sleep Wake Disorder" OR "Wake Disorder, Sleep" OR "Wake Disorders, Sleep Sleep Disorders" OR "Disorder, Sleep" OR "Disorders, Sleep" OR "Sleep Disorder" OR "Short Sleeper Syndrome" OR "Short Sleeper Syndromes" OR "Sleeper Syndrome, Short" OR "Sleeper Syndromes, Short" OR "Syndrome, Short Sleeper" OR "Syndromes, Short Sleeper" OR "Short Sleep Phenotype" OR "Phenotype, Short Sleep" OR "Phenotypes, Short Sleep" OR "Short Sleep Phenotypes" OR "Sleep Phenotypes, Short" OR "Sleep"  **AND Line 2**: "Polycystic Ovary Syndrome" OR "PCOS" OR "stein -leventhal Syndrome" OR "Sclerocystic Ovarian Degeneration" OR "Ovary Syndrome, Polycystic" OR "Syndrome, Polycystic Ovary" OR "Stein-Leventhal Syndrome" OR "Stein Leventhal Syndrome" OR "Syndrome, Stein-Leventhal" OR "Ovarian Degeneration, Sclerocystic" OR "Sclerocystic Ovary Syndrome" OR "Polycystic Ovarian Syndrome" OR "Ovarian Syndrome, Polycystic" OR "Polycystic Ovary Syndrome 1" OR "Sclerocystic Ovaries" OR "Ovary, Sclerocystic" OR "Sclerocystic Ovary" |
|  |
