# Supplementary material for: A meta-analysis of the relationship between polycystic ovary syndrome and sleep disturbances risk
Source: Front Physiol. 2022 Sep 29;13:957112. doi: 10.3389/fphys.2022.957112 (PMC9558285; doi:10.3389/fphys.2022.957112)
Supplement: Supplementary file 2 [file Table2.docx]

| **Section and Topic** | **Item #** | **Checklist item** | **Location where item is reported** |
| --- | --- | --- | --- |
| **TITLE** | | |  |
| Title | 1 | The report is identified as a meta-analysis | Page 1 |
| **ABSTRACT** | | |  |
| Abstract | 2 | To explore the relationship between polycystic ovary syndrome (PCOS) and the risk of Sleep disturbances by meta-analysis. | Page 2 |
| **INTRODUCTION** | | |  |
| Rationale | 3 | Sleep itself is an important regulator of endocrine function, and the endocrine system also plays an important role in regulating the sleep-wake cycle.There may be a complex relationship between PCOS as an endocrine disorder and sleep. Several studies have explored the relationship between PCOS and the risk of sleep disturbances. Due to the limited sample data, the results of each study are quite different, which has not only caused great trouble for clinicians, but also affected the prevention and treatment of Sleep disturbances in patients with PCOS. | Page 2-3 |
| Objectives | 4 | The purpose of this study is to conduct a meta-analysis of existing clinical studies so as to explore the relationship between PCOS and sleep disturbances, thereby providing evidence-based prevention and intervention for sleep disturbances in patients with medical evidence of PCOS. | Page 2-3 |
| **METHODS** | | |  |
| Eligibility criteria | 5 | Inclusion criteria: ① the subjects of the study were patients with a clinical diagnosis of PCOS in the case group, and healthy women with a normal menstrual cycle or female patients who came to the hospital for other reasons in the control group; ② the types of studies were cohort studies, case-control studies, and cross-sectional studies which were compared with the control group; ③ articles in which the subjects were diagnosed as having PCOS by the National Institutes of Health（NIH） criteria and Rotterdam criteria, and Sleep disturbances were diagnosed and investigated by polysomnography, actiwatch, and sleep questionnaires.  Exclusion criteria: ① languages other than English; ② replicated publications (if studies involving the same population were published repeatedly, the latest published studies or those with the larger sample size were selected); ③ studies without a control group; ③ studies for which the effect sizes could not be extracted or calculated; ④ studies with little research information or incomplete data and inconsistent outcome indicators; ⑤ studies on the use of contraceptives, metformin and other drugs that affect PCOS; ⑥ studies in which the population is accompanied by hypertension, diabetes, atypical adrenal 21-hydroxylase deficiency, androgen-secreting tumor, Cushing's syndrome, and any other disease that may cause Sleep disturbances; ⑦ the experimental group and control group had significant body mass index (BMI) differences (differences greater than 10 kg/m2 were excluded) which were likely to cause bias. | Page4-5 |
| Information sources | 6 | A comprehensive and systematic search was performed using the web databases of PubMed (updated to May 1st, 2022), Web of Science (updated to May 1st, 2022),Cochrane Library (updated to May 1st, 2022), and Embase (updated to May 1st, 2022). | Page 3 |
| Search strategy | 7 | The search terms included free words as well as MeSH subject headings. The English terms used to search the literature were as follows: "polycystic ovary syndrome","PCOS","Stein-leventhal Syndrome","Sclerocystic Ovarian Degeneration","Ovary Syndrome, Polycystic","Syndrome, Polycystic Ovary","Stein-Leventhal Syndrome","Stein Leventhal Syndrome","Syndrome, Stein-Leventhal","Ovarian Degeneration, Sclerocystic","Sclerocystic Ovary Syndrome","Polycystic Ovarian Syndrome","Sclerocystic Ovaries","Ovary, Sclerocystic","Sclerocystic Ovary”, Sleep Wake Disorders","Disorder, Sleep Wake" ,"sleep disturbances","Disorder, Sleep Wake","Disorders, Sleep Wake","Sleep Wake Disorder","Wake Disorder, Sleep","Wake Disorders, Sleep Sleep Disorders","Disorder, Sleep","Disorders, Sleep","Sleep Disorder","Short Sleeper Syndrome","Short Sleeper Syndromes","Sleeper Syndrome, Short","Sleeper Syndromes, Short","Syndrome, Short Sleeper","Syndromes, Short Sleeper","Short Sleep Phenotype","Phenotype, Short Sleep","Phenotypes, Short Sleep","Short Sleep Phenotypes","Sleep Phenotypes, Short","Sleep". The type of research design was not limited, and the language was limited to English. | Page 4 |
| Selection process | 8 | The quality of the articles was assessed by two researchers, and any disagreements were resolved through discussion until a consensus was reached or after consultation with the third author. | Page 5 |
| Data collection process | 9 | The three authors independently extracted the necessary data, and any disagreements were resolved through discussion until a consensus was reached or after consultation with the third author. The extraction of the relevant data from the included articles was then performed. | Page 5 |
| Data items | 10a | Data extracted from the literature included the first author, study area, publication time, sample size, age, BMI, prevalence of Sleep disturbances, AHI, sleep efficiency, rapid eye movement (REM) sleep, sleep onset latency, Pittsburgh Sleep Quality Index (PSQI), Epworth Sleepiness Scale (ESS), outcome measures, and adjustment for confounders.. | Page 5 |
|  | 10b | Table 1 Basic Characteristics of Included Articles | Table 1 |
| Study risk of bias assessment | 11 | As the included articles were case-control studies and cohort studies, NOS scale assessment was adopted. The lowest overall evaluation was 6★ and the highest was 8★, all of which were moderate to high quality. All studies had a low to moderate risk of bias and no studies were excluded due to poor quality (< 5★). | Page 6  Table 2 |
| Effect measures | 12 | Enumeration data was analyzed using odds ration (OR) and 95% confidence interval (CI). Data measurement was carried out by weighted mean difference (MD) and 95% CI, and P < 0.05 was considered statistically significant; the heterogeneity of the articles was evaluated through I^2^. If the included articles had no statistical heterogeneity (P > 0.01, I^2^ < 50%), the fixed effect model was used; if the included articles had statistical heterogeneity (P < 0.01, I^2^ > 50%), the random effect model was used, and sensitivity analysis was performed to understand. | Page 6 |
| Synthesis methods | 13a | Table 1 Basic Characteristics of Included Articles | Table 1 |
|  | 13b | For the included articles, RevMan 5.4.1 software provided by Cochrane was used for the meta-analysis of all statistical analyses. | Page 2 |
|  | 13c | The incidence of Sleep disturbances, PSQI score,ESS score,AHI,sleep efficiency,sleep onset latency,and REM sleep in the PCOS group and control group were used as outcome indicators. MD and OR values were used for effect evaluation, and 95% CI was calculated; the heterogeneity of the articles was evaluated through I^2^. If the included articles had no statistical heterogeneity (P > 0.01, I^2^ < 50%), the fixed effect model was used; if the included articles had statistical heterogeneity (P < 0.01, I^2^ > 50%), the random effect model was used, and sensitivity analysis was performed to understand the source of the heterogeneity. | Page 5-6 |
| Reporting bias assessment | 14 | The presence of publication bias was assessed using Egger’s method. There were four studies on the incidence of Sleep disturbances in the PCOS group and control group. The result of Egger’s method was P = 0.664 > 0.05, indicating no significant publication bias.There were four studies on the ESS score in the PCOS group and control group. The result of Egger’s method was P = 0.596 > 0.05, indicating no significant publication bias.There were four studies on the AHI in the PCOS group and control group. The result of Egger’s method was P = 0.427 > 0.05, indicating no significant publication bias.There were six studies on the Sleep Efficiency in the PCOS group and control group. The result of Egger’s method was P = 0.124 > 0.05, indicating no significant publication bias.There were four studies on the Sleep Onset Latency in the PCOS group and control group. The result of Egger’s method was P = 0.183 > 0.05, indicating no significant publication bias. | Page 10-11 |
| Certainty assessment | 15 | The effect evaluation of the incidence of the Sleep disturbances using the OR value, and 95% CI was calculated. MD values were used for the effect evaluation of the PCOS and PSQI score,PCOS and ESS score,PCOS and sleep efficiency,PCOS and sleep onset latency, PCOS and REM sleep,and 95% CI was calculated. | Page 7-9 |
| **RESULTS** | | |  |
| Study selection | 16a | Fig. 1 Process of Inclusion of Articles in Meta-analysis | Fig 1 |
| Study characteristics | 17 | Cite each included study and present its characteristics. | Table 1 |
| Risk of bias in studies | 18 | Present assessments of risk of bias for each included study. | Table 2 |
| Results of individual studies | 19 | 1.Association of PCOS with the incidence of Sleep disturbances.(OR = 11.24, 95% CI: 2.00–63.10, Z = 2.75, P = 0.006).  2.Association of PCOS with the PSQI score.(MD = 0.78, 95% CI: 0.32–1.25, Z = 3.30, P = 0.001).  3.Association of PCOS with the ESS score.(MD = 2.49, 95% CI: 0.80–4.18, Z = 2.88, P = 0.004).  4.Association of PCOS with the AHI events. (MD = 2.68, 95% CI: 1.07–4.28, Z = 3.27, P = 0. 001).  5.Association of PCOS with the sleep efficiency.(MD = -5.16, 95% CI: -9.39–-0.93, Z = 2.39, P = 0.02).  6.Association of PCOS with the sleep onset latency. (MD = 2.45, 95% CI: 1.40–3.50, Z = 4.57, P < 0.001).  7.Association of PCOS with the REM sleep. (MD = 17.19, 95% CI: 11.62–55.76, Z = 6.05, P < 0.001). | Page 7-9  Fig 2-6,8-9 |
| Results of syntheses | 20a | For each synthesis, briefly summarise the characteristics and risk of bias among contributing studies. | Page 7-9  Table 1 |
|  | 20b | Present results of all statistical syntheses conducted. If meta-analysis was done, present for each the summary estimate and its precision (e.g. confidence/credible interval) and measures of statistical heterogeneity. If comparing groups, describe the direction of the effect. | Page 7-9  Fig 2-6,8-9 |
|  | 20c | Present results of all investigations of possible causes of heterogeneity among study results. | Page 9-10 |
|  | 20d | Present results of all sensitivity analyses conducted to assess the robustness of the synthesized results. | Page 9-10 |
| Reporting biases | 21 | The presence of publication bias was assessed using Egger's method. | Page 10-11 |
| Certainty of evidence | 22 | Present assessments of certainty (or confidence) in the body of evidence for each outcome assessed. | Page 7-9 |
| **DISCUSSION** | | |  |
| Discussion | 23a | Provide a general interpretation of the results in the context of other evidence. | Page 11-13 |
|  | 23b | Discuss any limitations of the evidence included in the review. | Page 11-13 |
|  | 23c | Discuss any limitations of the review processes used. | Page 11-13 |
|  | 23d | Discuss implications of the results for practice, policy, and future research. | Page 14 |
| **OTHER INFORMATION** | | |  |
| Registration and protocol | 24 | **We are in the process of registering.(PROSPERO)** |  |
| Support | 25 | Describe sources of financial or non-financial support for the review, and the role of the funders or sponsors in the review. | Page 14 |
| Competing interests | 26 | Declare any competing interests of review authors. | Page 14 |

*From:*  Page MJ, McKenzie JE, Bossuyt PM, Boutron I, Hoffmann TC, Mulrow CD, et al. The PRISMA 2020 statement: an updated guideline for reporting systematic reviews. BMJ 2021;372:n71. doi: 10.1136/bmj.n71

For more information, visit: <http://www.prisma-statement.org/>
